# Combined milrinone and enteral metoprolol therapy in patients with septic myocardial depression

**DOI:** 10.1186/cc6976

**Published:** 2008-08-04

**Authors:** Christian A Schmittinger, Martin W Dünser, Maria Haller, Hanno Ulmer, Günter Luckner, Christian Torgersen, Stefan Jochberger, Walter R Hasibeder

**Affiliations:** 1 Department of Anaesthesiology and Critical Care Medicine, Innsbruck Medical University, Anichstrasse 35, 6020 Innsbruck, Austria; 2 Department of Anaesthesiology and Critical Care Medicine, Krankenhaus der Barmherzigen Schwestern, Schlossberg 1, 4910 Ried im Innkreis, Austria; 3 Department of Medical Biostatistics, Innsbruck Medical University, Schöpfstrasse 41/1, 6020 Innsbruck, Austria

## Abstract

**Introduction:**

The multifactorial etiology of septic cardiomyopathy is not fully elucidated. Recently, high catecholamine levels have been suggested to contribute to impaired myocardial function.

**Methods:**

This retrospective analysis summarizes our preliminary clinical experience with the combined use of milrinone and enteral metoprolol therapy in 40 patients with septic shock and cardiac depression. Patients with other causes of shock or cardiac failure, patients with beta-blocker therapy initiated more than 48 hours after shock onset, and patients with pre-existent decompensated congestive heart failure were excluded. In all study patients, beta blockers were initiated only after stabilization of cardiovascular function (17.7 ± 15.5 hours after shock onset or intensive care unit admission) in order to decrease the heart rate to less than 95 beats per minute (bpm). Hemodynamic data and laboratory parameters were extracted from medical charts and documented before and 6, 12, 24, 48, 72, and 96 hours after the first metoprolol dosage. Adverse cardiovascular events were documented. Descriptive statistical methods and a linear mixed-effects model were used for statistical analysis.

**Results:**

Heart rate control (65 to 95 bpm) was achieved in 97.5% of patients (n = 39) within 12.2 ± 12.4 hours. Heart rate, central venous pressure, and norepinephrine, arginine vasopressin, and milrinone dosages decreased (all *P *< 0.001). Cardiac index and cardiac power index remained unchanged whereas stroke volume index increased (*P *= 0.002). In two patients (5%), metoprolol was discontinued because of asymptomatic bradycardia. Norepinephrine and milrinone dosages were increased in nine (22.5%) and six (15%) patients, respectively. pH increased (*P *< 0.001) whereas arterial lactate (*P *< 0.001), serum C-reactive protein (*P *= 0.001), and creatinine (*P *= 0.02) levels decreased during the observation period. Twenty-eight-day mortality was 33%.

**Conclusion:**

Low doses of enteral metoprolol in combination with phosphodiesterase inhibitors are feasible in patients with septic shock and cardiac depression but no overt heart failure. Future prospective controlled trials on the use of beta blockers for septic cardiomyopathy and their influence on proinflammatory cytokines are warranted.

## Introduction

Septic cardiomyopathy refers to myocardial injury with or without lowered cardiac output in patients with sepsis [1,2]. In contrast to earlier beliefs concerning the frequency of septic cardiomyopathy, a recent prospective trial in 67 adult septic shock patients without previous cardiac disease reported an overall hypokinesia rate (left ventricular ejection fraction of less than 45%) of 60% [3]. As compared with patients able to maintain hyperdynamic circulation, survival is significantly compromised in septic shock patients with low systemic blood flow [4]. Even if cardiac output can be preserved, myocardial injury as indicated by increased plasma levels of troponin [5] or natriuretic peptides [6-8] is associated with poor outcome in septic shock.

The etiology of septic cardiomyopathy is multifactorial. Throughout the last decades, several pathogenetic mechanisms, including bacterial toxins, cytokines, nitric oxide, and reactive oxygen species, were identified [2,9]. Recently, the contributory role of adrenergic stress and catecholamine-induced toxicity has been suggested [2]. Similarities have been drawn between catecholamine-induced myocardial stunning [10,11] and septic cardiomyopathy [12]. Sepsis was found to be an important risk factor for development of the left ventricular apical ballooning syndrome [13], originally known as Takotsubo cardiomyopathy [14].

In view of the growing evidence for an association between beta adrenergic stress and the pathogenesis of septic cardiomyopathy [15], the administration of beta-blocking agents could be beneficial. Although at first glance it appears counterproductive to administer a potentially negative inotropic drug to a patient with myocardial depression, beta-blocker therapy improved myocardial oxygen utilization, decreased tumor necrosis factor-alpha production, and preserved cardiac function in a septic animal model [16]. Similarly, Gore and Wolfe [17] found that a continuous esmolol infusion reduced heart rate by 20% but did not compromise systemic oxygen delivery or organ blood flow in six hemodynamically stable patients with sepsis.

Apart from these studies, an increasing number of reports have been published suggesting advantageous effects of beta blockers in acute critical illness. Though recently challenged [18,19], perioperative beta blockade has repeatedly been shown to reduce cardiac complications and improve survival in high-risk surgery patients [20,21]. Similarly, preliminary data on the use of beta blockers in critically ill patients with severe trauma [22], traumatic brain injury [23], or burns [24] indicate a beneficial influence on morbidity and mortality.

In an effort to reduce tachycardia in patients with septic shock requiring inotropic therapy, we have cautiously started to use beta blockers. First, this therapeutic intervention was restricted to patients with chronic beta-blocker therapy in order to attenuate rebound tachycardia and decrease the risk of perioperative myocardial ischemia but later was also used in patients without chronic beta-blocker treatment in an attempt to decrease high heart rate and economize cardiac function. This retrospective analysis summarizes our preliminary clinical experience with the combined use of milrinone and enteral metoprolol therapy in 40 patients with septic shock and cardiac depression. Our hypothesis was that metoprolol would reduce heart rate without destabilizing cardiovascular function.

## Materials and methods

The retrospective protocol was approved by the Ethics Committee of the Krankenhaus der Barmherzigen Schwestern in Ried im Innkreis. In view of the retrospective study design, written informed consent was waived. From 1 January 2005 to 28 February 2008, all medical records of an eight-bed multidisciplinary intensive care unit (ICU) were reviewed for patients with the admission diagnosis of septic shock as defined by the American College of Chest Physicians and the Society of Critical Care Medicine [25]. All patients with septic shock and cardiac depression who were treated with enteral metoprolol within 48 hours after the onset of shock or admission to the ICU were included in the analysis. Cardiac depression was defined as a central venous oxygen saturation (ScvO_2_) of less than 65% despite adequate fluid resuscitation, oxygenation, and hematocrit, and/or a cardiac index (CI) of less than 2.5 L/minute per m^2 ^requiring inotropic therapy. Patients younger than 18 years, patients with any cause of low cardiac output other than sepsis (for example, myocardial ischemia), patients with pre-existent decompensated congestive heart failure (New York Heart Association classification III and IV), patients with septic shock who did not require inotropic support or in whom cardiac output was not measured, and patients who first received beta blockers more than 48 hours after the onset of shock or ICU admission were excluded.

### Hemodynamic and general treatment

**Figure 1:**
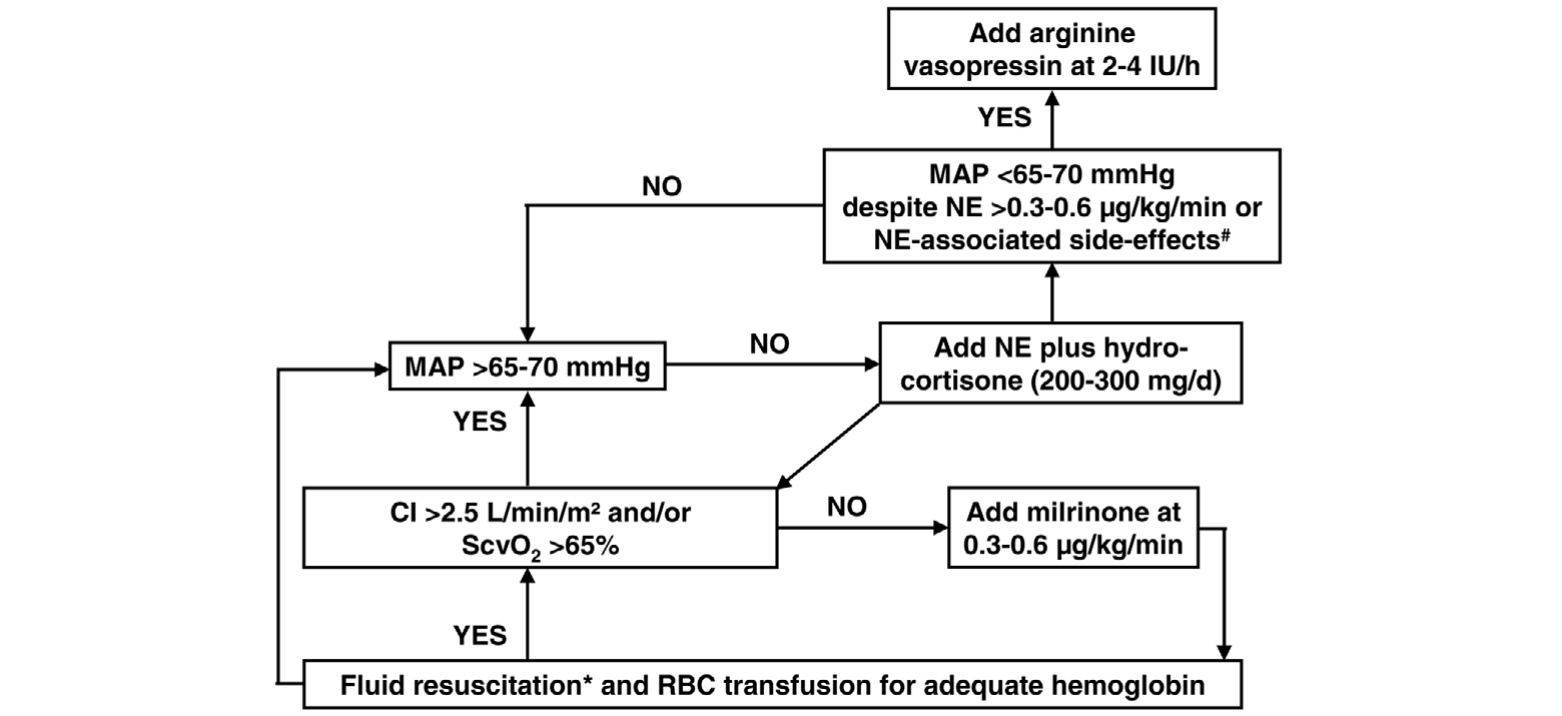
Institutional hemodynamic protocol. *Fluid resuscitation using crystalloids to cover basal fluid demands (~30 mL/kg per day) and colloids for further fluid loading (guided by responses in stroke volume and cardiac index, arterial and central venous pressure, heart rate, and clinical signs). Colloids hydroxyethyl starch (molecular weight, 130.000; Voluven^® ^130/0.4; Fresenius Kabi, Graz, Austria) with a dose limitation of 30 mL/kg per day based on the manufacturer's instructions and gelatine (molecular weight, 22.600; Gelofusin^®^; B. Braun, Melsungen, Germany) without a dose limitation were used. ^#^New-onset tachyarrhythmias, progressive tachycardia of greater than 110 beats per minute despite adequate fluid resuscitation, pulmonary arterial hypertension with new signs of right heart dysfunction, new-onset hyperglycemia (blood sugar of greater than 130 mg/dL) resistant to insulin dosages of greater than 5 IU/hour, new increase in troponin serum concentrations, or progressive deterioration of diastolic or systolic ventricular function. CI, cardiac index; MAP, mean arterial blood pressure; NE, norepinephrine; RBC, red blood cell; ScvO_2_, central venous oxygen saturation.

All septic shock patients were invasively monitored with an arterial and a central venous catheter as well as a transpulmonary thermodilution device to assess cardiac output (PICCO^®^; Pulsion Medical Systems, Munich, Germany). Hemodynamic resuscitation was performed according to an institutional protocol (Figure 1) that served as a recommendation for the attending physician. During shock, all patients were mechanically ventilated and sedated with a midazolam/fentanyl infusion. Continuous veno-venous hemofiltration with a minimum filtration rate of 35 mL/minute was commenced for renal indications only (n = 28, 70%). Nutrition was initiated via the parenteral route on ICU day 2 and gradually substituted with enteral nutrition starting on ICU day 3 or when cardiovascular function was stabilized.

### Beta-blocker therapy

In an effort to decrease the heart rate to less than 95 beats per minute (bpm), compassionate use of metoprolol was started as considered indicated by the physician in charge. First, metoprolol was restricted to patients with chronic beta-blocker therapy in order to attenuate rebound tachycardia and decrease the risk of perioperative myocardial ischemia, but after one third of the observation period was also used in patients without chronic beta-blocker treatment in an attempt to treat tachycardia and economize cardiac function. In all patients, beta blockers were initiated only after cardiovascular function had been stabilized. A retard formulation of metoprolol (Seloken retard^®^; AstraZeneca, Vienna, Austria) was used at 25 to 47.5 mg via the enteral route. Based on response in heart rate, stroke volume or CI, and arterial blood pressure, metoprolol was gradually increased to reach a targeted heart rate of 65 to 95 bpm. Metoprolol was transiently stopped or completely withdrawn if the heart rate dropped to less than 60 bpm.

### Data documentation

Where available, the following variables were extracted from routinely performed measurements documented in the medical charts: demographic data, pre-existent diseases, chronic beta-blocker therapy, source of infection, need for renal replacement therapy, length of stay in the ICU, and 28-day mortality. The Simplified Acute Physiology Score II [26] and a modified Goris multiple organ dysfunction syndrome score [27] were calculated from most aberrant clinical and laboratory variables during the first 24 hours after admission or during the ICU stay, respectively. Hemodynamic data were documented at shock onset and before and 6, 12, 24, 48, 72, and 96 hours after the first metoprolol dosage and included heart rate, mean arterial blood pressure (MAP), central venous pressure, ScvO_2_, CI, and stroke volume index (SVI) as well as norepinephrine, arginine vasopressin (AVP), and milrinone requirements. The cardiac power index (CPI), an index of cardiac contractility strongly correlated with outcome in acute and chronic heart failure [28], was calculated as the product of simultaneously measured MAP and CI (CPI [W/m^2^] = MAP × CI × 0.0022). Systemic vascular resistance was calculated according to the standard formula. Also documented were the time elapsed between shock onset and initiation of metoprolol therapy as well as the time between initiation of metoprolol therapy and attainment of the targeted heart rate range. Serum concentrations of creatinine, aspartate, and alanine aminotransferase, total bilirubin, troponin I, C-reactive protein, and the arterial oxygen tension/inspiratory oxygen tension (PaO_2_/FiO_2_) quotient were recorded before and 24, 48, 72, and 96 hours after the start of metoprolol. pH and arterial lactate levels were documented before and 6, 12, 24, 48, 72, and 96 hours after the first metoprolol dosage.

### Definition of adverse events

To evaluate the incidence of adverse events during the 96-hour observation period, the following definitions were retrospectively applied. A decrease in arterial blood pressure was considered a greater than 20% reduction in MAP as compared with baseline values or an MAP of less than 65 mm Hg at two or more time points, both requiring an increase in norepinephrine support. A decrease in CI, SVI, or ScvO_2 _was similarly defined as a greater than 20% reduction as compared with baseline values at two or more time points, requiring an increase in inotropic support and/or withdrawal of metoprolol therapy. Bradycardia was defined as a drop in heart rate to less than 60 bpm and was considered to be symptomatic if it resulted in an MAP of less than 65 mm Hg or CI of less than 2.5 L/minute per m^2^.

### Statistical analysis

The primary endpoint was to assess the clinical course and hemodynamic parameters during combined milrinone and metoprolol therapy. The secondary endpoint was to evaluate changes in laboratory and organ function parameters. The SPSS^® ^12.0.1. software package was used for statistical analysis (SPSS Inc., Chicago, IL, USA). Kolmogorov-Smirnov tests were used to verify normal distribution of study variables, which was approximately given for all variables except serum liver enzymes and total bilirubin concentrations, as well as arterial lactate levels. After ln transformation, the normality assumption was achieved for these variables, too. Descriptive statistical methods were applied to present demographic and clinical data and to evaluate the incidence of adverse events. Changes in hemodynamic or laboratory parameters during metoprolol therapy were assessed with a linear mixed-effects model. In contrast to conventional tests such as the analysis of variance, this method can evaluate changes over time despite the fact that some patients dropped out because they died during the observation period [29]. If changes over time were significant, comparisons versus baseline values were performed using the same model and applying Bonferroni corrections. *P *values of less than 0.05 were considered to indicate statistical significance. All variables are given as mean value ± standard deviation, if not indicated otherwise.

## Results

During the review period, 174 patients with septic shock were treated in the ICU. Forty of those patients were treated with a milrinone infusion and received enteral metoprolol during the first 48 hours after shock onset or ICU admission (17.7 ± 15.5 hours) (Table 1). Seven patients died during the observation period. Heart rate was reduced to the targeted range of 65 to 95 bpm in 97.5% of the patients (n = 39) within 12.2 ± 12.4 hours. Of the 36 patients treated with AVP, 31 received AVP before baseline measurements whereas 5 patients were started on AVP therapy during the observation period (within 6 hours, n = 4; within 24 hours, n = 1). While heart rate and central venous pressure significantly decreased, SVI increased during the observation period. At the same time, norepinephrine, AVP, and milrinone dosages were significantly reduced (Table 2). A significant increase in pH as well as decreases in arterial lactate, serum creatinine, and C-reactive protein levels were seen during the observation period (Table 3). Metoprolol therapy was discontinued in two patients because asymptomatic bradycardia occurred and heart rate remained within the lower targeted limits after one and two metoprolol dosages, respectively. The incidence of adverse events during the observation period is presented in Table 4.

**Table 1:** Characteristics of the study population

Number	40
Age, years	71 ± 13
Male gender, number (percentage)	21 (53)
Body mass index, kg/m^2^	28 ± 7
Premorbidities, number (percentage)	
Chronic arterial hypertension	23 (58)
Obstructive coronary artery disease	10 (25)
Compensated congestive heart failure	12 (30)
Chronic obstructive pulmonary disease	8 (20)
Chronic renal insufficiency	14 (35)
Chronic liver disease	4 (10)
Neoplasm	3 (8)
Chronic beta-blocker therapy, number (percentage)	15 (38)
Source of infection, number (percentage)	
Liver/Abdomen	21 (53)
Lung	10 (25)
Skin/Soft tissue	3 (8)
Joint/Bone	2 (5)
Catheter/Device	1 (3)
Urogenital tract	1 (3)
Unknown origin	2 (5)
Continuous veno-venous hemofiltration, number (percentage)	28 (70)
Multiple organ dysfunction syndrome score (12), points	9.9 ± 2.3
Simplified Acute Physiology Score II, points	53 ± 16
Intensive care unit length of stay, days	15 ± 11
28-day mortality, number (percentage)	13 (33)

Data are presented as mean value ± standard deviation, if not indicated otherwise.

**Table 2:** Hemodynamic variables at shock onset and during the observation period

	ICU admission^a^	Baseline	6 hours	12 hours	24 hours	48 hours	72 hours	96 hours	*P *value
Patients, number	40	40	40	39	37	37	35	33	
Heart rate, bpm	110 ± 19	101 ± 18	84 ± 17^b^	84 ± 14^b^	84 ± 13^b^	83 ± 13^b^	79 ± 13^b^	78 ± 14^b^	<0.001^c^
MAP, mm Hg	59 ± 19	85 ± 23	82 ± 15	85 ± 18	87 ± 15	90 ± 20	91 ± 20	90 ± 21	0.16
CVP, mm Hg	14 ± 4	12 ± 3	12 ± 4	12 ± 3	11 ± 3	11 ± 3^b^	10 ± 3^b^	9 ± 3^b^	<0.001^c^
Cardiac index, L/minute per m^2^	1.9 ± 0.6	3.1 ± 1.1	3.2 ± 1.0	3.3 ± 0.9	3.4 ± 0.9	3.4 ± 1.0	3.5 ± 1.0	3.5. ± 0.8	0.56
SVI, mL/beat per m^2^	18 ± 7	32 ± 12	40 ± 14	40 ± 12	42 ± 12^b^	42 ± 13^b^	42 ± 10^b^	44 ± 9^b^	0.002^c^
CPI, W/m^2^	0.24 ± 0.14	0.61 ± 0.32	0.57 ± 0.22	0.60 ± 0.17	0.65 ± 0.18	0.68 ± 0.30	0.71 ± 0.25	0.68 ± 0.23	0.27
ScvO_2_, percentage	64 ± 12	71 ± 10	72 ± 6	72 ± 11	74 ± 9	77 ± 8	73 ± 11	72 ± 11	0.35
SVRI, dyne-second/cm^5 ^per m^2^	2,041 ± 1,181	2,114 ± 825	1,918 ± 897	1,913 ± 777	1,895 ± 647	2,014 ± 800	2,060 ± 852	1,824 ± 569	0.78
NE, μg/kg per minute	0.12 ± 0.25 (n = 18)	0.17 ± 0.11	0.18 ± 0.11	0.18 ± 0.11	0.17 ± 0.13	0.13 ± 0.13	0.09 ± 0.08^b^	0.06 ± 0.07^b^	<0.001^c^
AVP dosage, IU/hour	NA	2.0 ± 1.6	2.2 ± 1.3	2.1 ± 1.3	2.1 ± 1.2	1.9 ± 1.3	1.3 ± 1.3	0.8 ± 1.1^b^	<0.001^c^
Mil, μg/kg per minute	0.24 ± 0.19 (n = 6)	0.31 ± 0.16	0.34 ± 0.17	0.33 ± 0.16	0.30 ± 0.17	0.24 ± 0.18	0.21 ± 0.19	0.12 ± 0.13^b^	<0.001^c^
Meto, mg	NA	47 ± 19	NA	NA	47 ± 41	52 ± 42	51 ± 42	54 ± 37	NA

^a^Not included in the longitudinal mixed-effects analysis. ^b^Significant effects versus baseline. ^c^Significant time effect. Data are presented as mean value ± standard deviation. AVP, arginine vasopressin; bmp, beats per minute; CPI, cardiac power index; CVP, central venous blood pressure; ICU, intensive care unit; MAP, mean arterial blood pressure; Meto, metoprolol dosage; Mil, milrinone requirements (n in parentheses indicates the number of patients who received milrinone already at intensive care unit admission); NA, not administered; NE, norepinephrine requirements (n in parentheses indicates the number of patients who received norepinephrine already at intensive care unit admission); ScvO_2_, central venous oxygen saturation; SVI, stroke volume index; SVRI, systemic vascular resistance index.

**Table 3:** Organ function variables during the observation period

	Baseline	6 hours	12 hours	24 hours	48 hours	72 hours	96 hours	*P *value
Patients, number	40	40	39	37	37	35	33	
pH	7.36 ± 0.09	7.37 ± 0.06	7.37 ± 0.1	7.38 ± 0.08	7.38 ± 0.07^a^	7.4 ± 0.06^a^	7.42 ± 0.07^a^	<0.001^b^
Lactate, mg/dL	22 ± 15	24 ± 14	29 ± 32	14 ± 10^a^	12 ± 8^a^	11 ± 7^a^	10 ± 5^a^	<0.001^b^
Creatinine, mg/dL	2.3 ± 1.3	NM	NM	2.0 ± 1.0	1.8 ± 0.7	1.7 ± 0.8	1.6 ± 0.7^a^	0.02^b^
ASAT, IU/L	230 ± 651	NM	NM	143 ± 253	166 ± 320	199 ± 474	153 ± 336	0.97
ALAT, IU/L	128 ± 435	NM	NM	78 ± 222	90 ± 225	101 ± 207	90 ± 157	0.78
Bilirubin, mg/dL	1.7 ± 1.4	NM	NM	1.6 ± 1.3	1.5 ± 1.1	1.5 ± 1.5	1.6 ± 2.2	0.60
C-reactive protein, mg/dL	17.6 ± 8.7	NM	NM	17.8 ± 9.1	15.2 ± 9.3	11.6 ± 8.6	10 ± 8.2	0.001^b^
Troponin I, μg/L	8 ± 40	NM	NM	6 ± 21	3 ± 9	3 ± 7	2 ± 5	0.60
Platelet count, 10^9^/L	145 ± 78	NM	NM	132 ± 88	130 ± 106	134 ± 112	133 ± 123	0.95
PaO_2_/FiO_2_	244 ± 129	NM	NM	243 ± 92	252 ± 102	238 ± 84	262 ± 89	0.87

^a^Significant effects versus baseline. ^b^Significant time effect. Data are presented as mean value ± standard deviation. ALAT, alanine aminotransferase; ASAT, aspartate aminotransferase; FiO_2_, inspiratory oxygen tension; NM, not measured; PaO_2_, arterial oxygen tension.

**Table 4:** Adverse events during the observation period

	Number (percentage)
Asymptomatic bradycardia	2 (5)
Symptomatic bradycardia	0 (0)
Increase in norepinephrine dosage	9 (22.5)
Decrease in cardiac index	7 (17.5)
Decrease in cardiac index and ScvO_2_	1 (2.5)
Decrease in stroke volume index	2 (5)
Increase in milrinone dosage	6 (15)

ScvO_2_, central venous oxygen saturation.

## Discussion

After cardiovascular stabilization, heart rate and central venous pressure decreased and SVI increased in this study population during combined milrinone and enteral metoprolol therapy. Simultaneously, vasopressor and inotropic drug support was reduced. Except for an increase in pH as well as decreases in arterial lactate, serum creatinine, and C-reactive protein levels, organ function variables remained unchanged.

Pathophysiologically, septic cardiomyopathy is defined as an inadequately increased cardiac output in relation to the lowered systemic vascular resistance in sepsis and does not necessarily imply that cardiac output is absolutely decreased [1,2]. Indeed, overt cardiac failure as known from patients with cardiogenic shock is rare and has been observed in a maximum of 10% to 15% of septic shock patients [30]. More commonly, the clinical picture of septic cardiomyopathy is characterized by a variable degree of myocardial depression which can be detected echocardiographically or biochemically through elevated troponin levels in greater than or equal to 50% [3,31] and greater than 40% [5], respectively, of sepsis patients. Independently of the presence of overt cardiac failure, the grade of myocardial depression correlates with poor prognosis in sepsis [2,4]. In our analysis, all patients suffered from septic shock with considerably impaired cardiac pump function requiring infusion of an inotropic agent. Even though moderately elevated troponin I serum concentrations in 92.5% of the study patients (n = 37) further underline the presence of septic cardiomyopathy, the lack of echocardiography data limits the detailed investigation of cardiac dysfunction in our analysis.

Despite the growing evidence that beta blockers can be safely and probably beneficially administered in acute critical illness, current use of beta-blocking agents in patients with septic shock and cardiomyopathy must definitely be considered experimental. In an attempt to reduce tachycardia in patients with chronic beta-blocker therapy in whom rebound tachycardia was suspected [32], we have initiated enteral metoprolol therapy for the first time. The results of a study of the favorable effects of perioperative beta blockade [33] then prompted us to employ beta blockers in patients without previous chronic beta-blocker therapy. Nonetheless, in all patients, this was done compassionately and as considered indicated by the treating physician, starting cautiously with low metoprolol dosages and under tight control of cardiac output and ScvO_2_.

A selective beta-1, instead of a non-selective, beta blocker was chosen to prevent inhibition of potentially beneficial beta-2 effects [34]. Since in our clinical experience esmolol infusion was associated with frequent and rapid decreases in heart rate and cardiac output, metoprolol was applied via the enteral route. Accordingly, Gore and Wolfe [17] observed a 20% decrease in CI during esmolol infusion in hemodynamically stable sepsis patients. Since heart rate is a major determinant of myocardial oxygen consumption [35], it was used to dose metoprolol. In cardiovascular high-risk patients, a heart rate of 95 bpm was shown to be the critical threshold at which myocardial oxygen demand outstripped coronary supply and myocardial ischemia was likely to occur [36,37].

Instead of beta-agonists, a phosphodiesterase III inhibitor was applied as an inotropic agent in all of our patients. Although this does not correspond to current recommendations [38], milrinone has been used in patients with septic shock at our institution throughout the last decade. Positive inotropic effects of milrinone are mediated through inhibition of the breakdown of cAMP by phosphodiesterases [39] and act independently of beta-1 receptors. In view of differences in cAMP-independent actions [40] and compartmentation of cAMP-mediated signaling [41], the combination of milrinone and metoprolol may hold potential benefits for myocardial function [39].

A decrease in heart rate together with an increase in SVI given an unchanged CI can be interpreted as an economization of cardiac work and oxygen consumption. Reduced heart rates lower the risk of myocardial ischemia [21,36,37], particularly in patients with obstructive coronary artery disease [42]. In light of diminishing milrinone support, these observations may even reflect improved cardiac pump function in our study patients. Moreover, a decrease in central venous pressure as observed during metoprolol therapy often follows amelioration of myocardial performance [28]. The observation that organ function variables remained unchanged during beta-blocker therapy strengthens the assumption that metoprolol therapy did not reduce systemic blood flow or limit organ oxygen supply. Similarly, organ blood flow (extremity and hepatic blood flow) was not overtly affected during esmolol infusion in six hemodynamically stable sepsis patients [17]. Since metoprolol therapy was commenced 17.7 ± 15.5 hours after the onset of shock and initiation of standard hemodynamic therapy, it is unlikely that cardiovascular changes simply resulted from fluid therapy, vasopressor, or milrinone infusion. However, because of the uncontrolled design, our study cannot prove a causative relationship between the observed hemodynamic changes and metoprolol therapy.

In view of the preference of milrinone over dobutamine and the frequent use of a supplementary AVP infusion, the hemodynamic effect during enteral metoprolol therapy can be interpreted only in the context of our institutional hemodynamic protocol. For example, infusion of AVP in 95.6% of the study patients could have interfered with the hemodynamic effects of metoprolol therapy. Likewise, a decrease in heart rate as well as a mild increase in CI was reported during supplementary AVP infusion in patients with advanced vasodilatory shock and hypodynamic circulation [43]. Although in most patients (89%) AVP was started before metoprolol therapy, we cannot determine the extent to which this influenced the hemodynamic course during the observation period.

Response to metoprolol in this study population was not entirely homogeneous. Whereas overall MAP and CI did not decrease, nine and seven patients exhibited a decrease in MAP and CI, respectively, requiring an increase in norepinephrine or milrinone dosages during the observation period. It cannot be proven that the observed changes reflect beneficial or adverse effects of metoprolol. It is conceivable that without beta-blocker therapy SVI would have increased even more and milrinone infusion could have been withdrawn earlier. Similarly, the decrease in MAP and CI in some patients may have resulted from the course of the underlying disease process instead of being related to metoprolol therapy.

Data from experimental and clinical studies suggest that several beta-blocker effects such as heart rate control [44], antagonization of catecholamine-induced stunning of the myocardium [11,45,46], and reduction of myocardial inflammation [47,48] may be beneficial in patients with septic myocardial depression. Although data are still conflicting [49], beneficial effects of beta blockers were reported to also include attenuation of an overshooting immune response. Adult trauma patients treated with a continuous beta-blocker infusion exhibited lower serum interleukin-6 levels than did controls receiving standard of care [22]. Reduced proinflammatory cytokine production was also illustrated during esmolol infusion in rats with septic cardiomyopathy [16]. Interestingly, serum C-reactive protein levels decreased during metoprolol therapy in our study. Although it could be hypothesized that metoprolol reduced interleukin-6 levels and thus C-reactive protein levels [50], many other factors such as focus control [38], adequate antibiotic therapy [38], and hemodynamic stabilization [51] are likely to have caused the decrease of C-reactive protein levels in our analysis.

In addition to the uncontrolled study design, other important limitations need to be noted when interpreting the results of our analysis. First, this is a retrospective study and it entails potential difficulties because of missing values in individual patients. Furthermore, patient enrolment was not performed according to a strict protocol as in a prospective trial but at the discretion of the attending physician. Therefore, some patients who would have been eligible for metoprolol therapy according to our treatment scheme may have been missed. Second, although a hemodynamic protocol that served as a recommendation for resuscitation of septic shock patients was available, we cannot be sure whether the attending physicians strictly adhered to the protocol during resuscitation of all study patients. Third, a population of 40 patients is too small for adequately evaluating the safety profile of metoprolol therapy in patients with septic shock and cardiac depression.

## Conclusion

Low doses of enteral metoprolol in combination with phosphodiesterase inhibitors are feasible in patients with septic shock and cardiac depression but no overt heart failure. Future prospective controlled trials on the use of beta blockers for septic cardiomyopathy and their influence on proinflammatory cytokines are warranted.

## Key messages

• Heart rate significantly decreased during combined milrinone infusion and enteral metoprolol therapy in patients with septic shock and cardiac depression.

• In 97.5% of patients, targeted heart rates of 65 to 95 beats per minute were achieved.

• Enteral metoprolol therapy appears to have no major adverse effects on cardiovascular or organ function.

• Mean arterial blood pressure increased despite decreasing norepinephrine, arginine vasopressin, and milrinone dosages.

• Cardiac function economized, resulting in a maintained cardiac index with a lower heart rate and a higher stroke volume index.

## Abbreviations

AVP = arginine vasopressin; bpm = beats per minute; CI = cardiac index; CPI = cardiac power index; ICU = intensive care unit; MAP = mean arterial blood pressure; ScvO_2 _= central venous oxygen saturation; SVI = stroke volume index.

## Competing interests

The authors declare that they have no competing interests.

## Authors' contributions

CAS made substantial contributions to the acquisition, analysis, and interpretation of data and was involved in drafting the manuscript. MWD made substantial contributions to the concept and design of the study and the acquisition, analysis, and interpretation of data and was involved in drafting the manuscript. MH, GL, CT, and SJ made substantial contributions to the acquisition of data and critically revised the manuscript for important intellectual content. HU performed the statistical analysis and critically revised the manuscript for important intellectual content. WRH made substantial contributions to the concept and design of the study and was involved in drafting the manuscript. All authors read and approved the final manuscript.
